# Bacteriophage T4 Head: Structure, Assembly, and Genome Packaging

**DOI:** 10.3390/v15020527

**Published:** 2023-02-14

**Authors:** Venigalla B. Rao, Andrei Fokine, Qianglin Fang, Qianqian Shao

**Affiliations:** 1 Bacteriophage Medical Research Center, Department of Biology, The Catholic University of America, Washington, DC 20064, USA; 2 Department of Biological Sciences, Purdue University, West Lafayette, IN 47907, USA; 3 School of Public Health (Shenzhen), Sun Yat-sen University, Shenzhen 518107, China

**Keywords:** bacteriophage T4, head assembly, ATPase motor, portal vertex, DNA packaging

## Abstract

Bacteriophage (phage) T4 has served as an extraordinary model to elucidate biological structures and mechanisms. Recent discoveries on the T4 head (capsid) structure, portal vertex, and genome packaging add a significant body of new literature to phage biology. Head structures in unexpanded and expanded conformations show dramatic domain movements, structural remodeling, and a ~70% increase in inner volume while creating high-affinity binding sites for the outer decoration proteins Soc and Hoc. Small changes in intercapsomer interactions modulate angles between capsomer planes, leading to profound alterations in head length. The in situ cryo-EM structure of the symmetry-mismatched portal vertex shows the remarkable structural morphing of local regions of the portal protein, allowing similar interactions with the capsid protein in different structural environments. Conformational changes in these interactions trigger the structural remodeling of capsid protein subunits surrounding the portal vertex, which propagate as a wave of expansion throughout the capsid. A second symmetry mismatch is created when a pentameric packaging motor assembles at the outer “clip” domains of the dodecameric portal vertex. The single-molecule dynamics of the packaging machine suggests a continuous burst mechanism in which the motor subunits adjusted to the shape of the DNA fire ATP hydrolysis, generating speeds as high as 2000 bp/s.

## 1. Introduction

One of us (Rao) was closely associated with Lindsay Black for 4 decades. The following are a few words from him on this background: my journey with Lindsay began on 7 December 1980, on a blistering cold winter night in Baltimore. It was fitting that I came to Lindsay’s lab “cold”, knowing little about bacteriophage T4 or phages in general. I was a biochemist by training, worked on fungal amylases for my PhD at the Indian Institute of Science, Bangalore, and took the first flight to US the day after submitting my thesis. Lindsay picked me up at the airport and drove me in his ancient stick-shift Volkswagen Beetle and dropped me off in a dorm room across his laboratory at the University of Maryland Medical School, Baltimore. So it began.

For the next 9 years, Lindsay’s trust, extraordinary patience, unique insights, and bedrock principle of empowering his students and postdocs fueled my passion for T4 phage and led to our first biochemical papers on DNA packaging [1,2]. We continued sharing our interests over the next 30-plus years and cowrote several review articles on T4 head structure, assembly, and genome packaging [3,4,5]. What follows here is an update of these reviews, focusing on the most significant recent advancements and discoveries of these shared interests. 

## 2. Architecture of T4 Head

The T4 and T4-like bacteriophages belong to *Straboviridae* family and are ubiquitously distributed on Earth [6,7]. They occupy nearly all environmental niches that are often quite hostile, such as the guts of mammals and humans, and sewage waters. Phage T4 infecting the *Escherichia coli* bacterium has long served as an extraordinary model to elucidate the basic mechanisms of molecular biology. Its particular similarities with herpes viruses [8] make it a compelling model to tease out the mechanisms of virus assembly and genome packaging, and to develop as a platform for drug discovery, vaccine design, and other biotechnology and biomedical applications [9,10,11].

**Figure 1:**
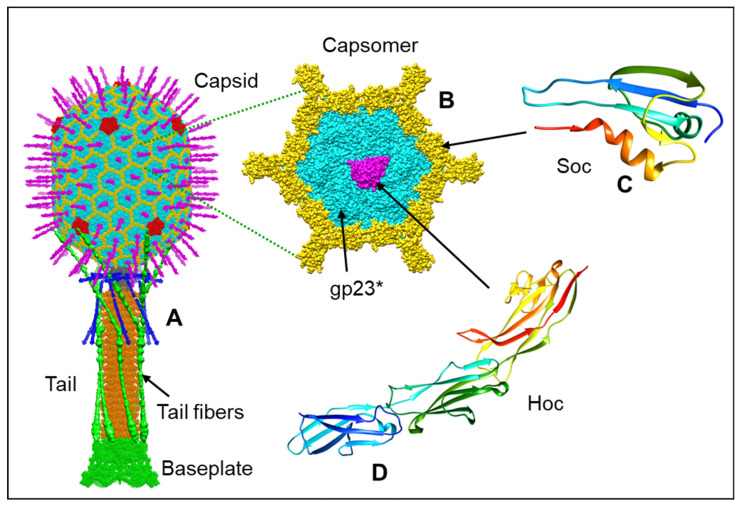
Structural model of bacteriophage T4 virion. The bacteriophage T4 virion (**A**) has a 120 × 86 nm prolate capsid packaged with ~171 kb linear double-stranded genomic DNA. The major capsid protein, gp23*, is shown in cyan; the vertex protein, gp24*, in red; the Soc protein in yellow; and the Hoc protein in magenta. A ~140 nm contractile tail with a baseplate is attached to the head through a dodecameric portal-neck connector. Six ~160 nm long tail fibers emanating from the baseplate are shown here in the “up” position. One of the hexameric capsomers (**B**) is enlarged to depict the arrangement of gp23*, Soc, and Hoc subunits. The atomic models of Soc (**C**) (PDB ID 5VF3) and of Hoc three Ig-like domains (**D**) (PDB ID 3SHS) are shown in rainbow colors.

The basic features of T4 virion include a large elongated (prolate) icosahedral head containing 155 hexameric capsomers made of the major capsid protein gp23* and 11 pentameric vertices made of the minor capsid protein gp24* (Figure 1A,B). The 12th vertex is a unique dodecameric portal vertex made of gp20 through which the genome enters and exists the capsid. T4 has a 140 nm long contractile tail, which terminates with a complex multiprotein hexagonal baseplate to which six ~160 nm long kinked tail fibers are attached. The T4 head, tail, and fibers assemble by separate pathways and then join to form the infectious virion [12,13]. 

### 2.1. Capsid Shell

**Figure 2:**
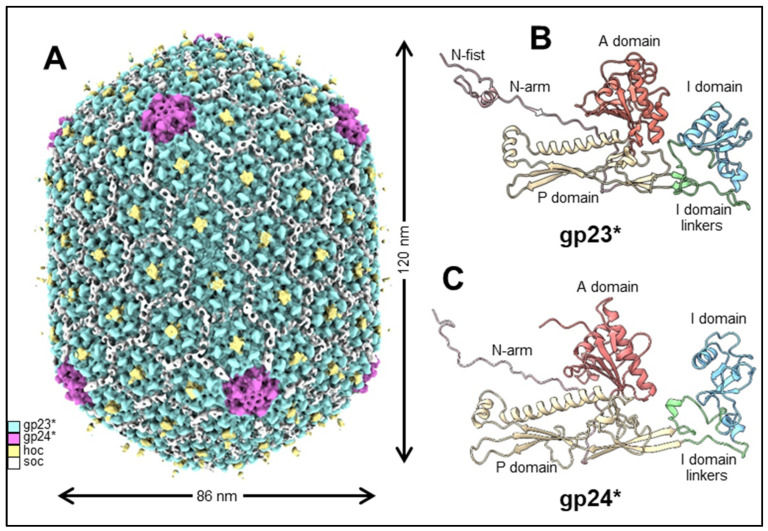
Structure of the mature phage T4 prolate head and the atomic models of gp23* and gp24*. (**A**) Cryo-EM structure of the phage T4 prolate head (EMD-6323). Ribbon diagrams of gp23* (**B**) and gp24* (**C**) (PDB ID: 5VF3) depicting the HK97 fold and the key subdomains.

The structure and dimensions of the phage T4 prolate capsid shell are displayed in Figure 2A. The head is elongated along its fivefold axis and has a length of 120 nm and a width of 86 nm [14,15]. The head encapsidates ~171 kbp linear double-stranded genomic DNA (~2–3% more than the unit-length genome). The major capsid protein, gp23*, is organized into a hexagonal lattice characterized by the triangulation numbers T_end_ = 13 *laevo* for the end caps and T_mid_ = 20 for the elongated midsection [14]. Gp23* is the cleaved form of gp23 from which the 65 N-terminal residues are removed during capsid maturation by a prohead protease. The prolate shell contains 930 subunits, or 155 hexameric capsomers of gp23* [14,16,17]. 

Like the major capsid proteins of other tailed phages, gp23* subunits [15,18] have a polypeptide fold similar to that of the bacteriophage HK97 capsid protein [19] (Figure 2B). This fold is characterized by the wedge-shaped axial (A) domain located near the capsomer axis and the peripheral (P) domain, forming the capsomer’s periphery [19]. T4 gp23* has an additional 60-residue globular insertion (I) domain, which makes characteristic bumps on the capsid surface [15,18,20,21]. This I domain is connected to the rest of the structure via long linkers, which are analogous to the elongated E loop in the HK97 fold [19]. The I domain is involved in extensive intra-capsomer interactions [15,18]. In the gp23* capsomers, the I domain sits on top of a neighboring subunit belonging to the same hexameric capsomer, thus greatly reinforcing the capsomer structure. The I domain linkers are also involved in stabilizing interactions with the subunits of the same capsomer and with neighboring capsomers. Although extra domains inserted into the HK97 fold were also observed in the capsid proteins of other phages, such as phi29 and P22 [22,23,24,25], the T4 I domain inserted into the E loop and resting on a neighboring subunit from the same capsomer is a unique feature of T4-like phages.

In the mature capsid, the gp23* protein contains an extended N-arm in its N-terminal region, like the major capsid protein of HK97. In addition, gp23* contains an unusual 25-residue N-terminal N-fist (Figure 2B) structure that interacts with four subunits: two from the same capsomer and two from an adjacent capsomer [18].

The intercapsomer binding is reinforced by attractive electrostatic forces between the P domains of gp23* subunits in different capsomers. Specifically, the negatively charged small helix from one P domain interacts with a positively charged β-sheet from an adjacent P domain [15,18]. These electrostatic interactions occur near the quasi-threefold axes that relate adjacent capsomers. Electrostatic interactions between the similar regions of the capsid proteins were observed in other phages and are conserved largely in the immature (unexpanded) and mature (expanded) capsid structures (see below). Consequently, a large network of extensive intra- and intercapsomer interactions form, generating a stable capsid structure capable of withstanding the substantial internal pressure (~25 atm) induced by the tightly packed genomic DNA [26,27]. 

Further, 11 of the 12 vertices of the capsid are occupied by pentamers of the gp24* protein, the cleaved form of gp24 lacking the first 10 residues removed by prohead protease during maturation. The high-resolution structures of the T4 capsid show that the gp24* structure is quite similar to gp23* [15,18,20] (Figure 2C), although the sequence identity between these two proteins is only ~20%. Thus, contrary to other well-studied phages, in which the major capsid protein occupies the pentameric vertices, T4 has evolved a separate gp24* protein specifically tailored to make the vertices, regions of the capsid where the curvature is the highest. The network of interaction observed between gp24* and neighboring subunits is similar to that observed for gp23*, though the gp24* structure is adapted for the vertex environment [15,18]. 

The gp24 protein is essential for phage viability. However, point mutations that bypass the gp24 requirement were found in gene 23 [16,28]. These mutations allow gp23 to substitute for gp24 in the vertices when gp24 is absent. Some of the gp24 bypass mutations sites are in the A domain of gp23, in the interface between adjacent subunits of the same capsomer. These mutations probably alter the gp23 structure and allow it to make pentamers (in addition to the usual hexamers) that now occupy the pentameric vertices. The vertex protein gp24 was probably derived from gp23 by gene duplication, followed by sequence divergence and optimization to adjust to its specific role of making stable pentameric vertices. On the other hand, the wild-type major capsid protein gp23, while optimized for hexamer assembly, probably lost its ability to assemble stable pentameric vertices during evolution, but this feature can be restored by the gp24 bypass mutations.

### 2.2. Portal

**Figure 3:**
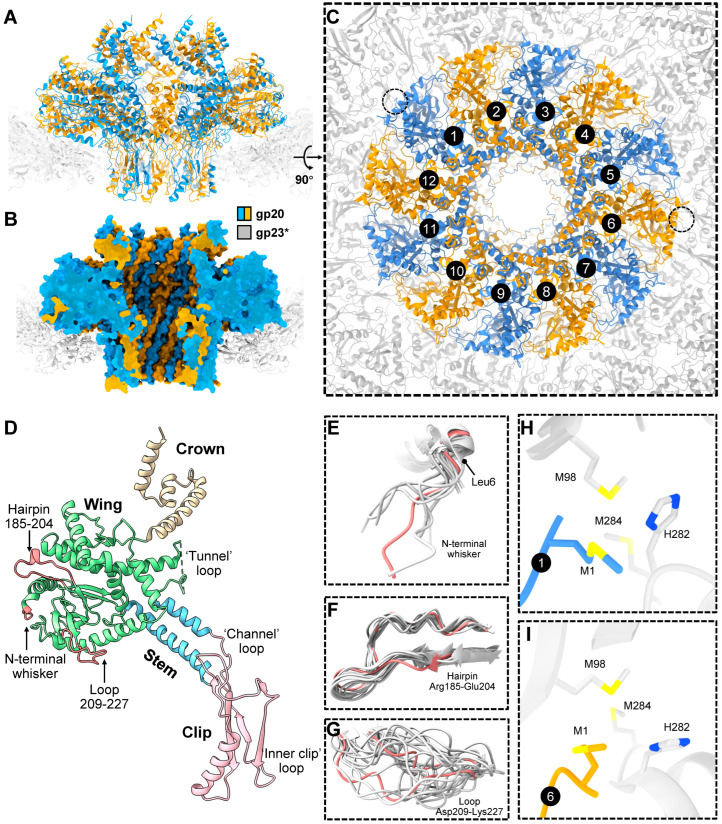
Structures of the portal protein gp20 and the symmetry-mismatched portal vertex. (**A**–**C**) Side views and top view of the portal protein assembly and its surrounding capsomers (PDB ID: 6UZC). The molecular surface of the portal protein assembly is shown in panel B to display the central channel. The 12 gp20 subunits are alternatively colored orange and blue and are labeled from 1 to 12 in panel (**C**). The gp23* subunits are colored gray. (**D**) Ribbon diagram of subunit 1, depicting the subdomains of the portal protein gp20. (**E**–**G**) Structural comparisons of the N-terminal whisker (panel (**E**)), hairpin Arg185-Glu204 (panel (**F**)), and loop Asp209-Lys227 (panel (**G**)) among the 12 gp20 subunits within the portal protein assembly. (**H**,**I**) Enlarged views of circled regions in panel (**C**), showing the two potential metal clusters between gp20 subunit 1 and its neighboring gp23* subunits (panel (**H**)) and between gp20 subunit 6 and its neighboring gp23* subunits.

In addition to the 11 pentameric vertices occupied by gp24*, the T4 capsid, like most icosahedral phages and herpes viruses, has a unique portal vertex that creates a platform for attaching the “neck” and tail. This vertex is occupied by a dodecamer of the portal protein, gp20, which initiates the capsid assembly [4,16,29] and also creates the binding site for the DNA-packaging motor [30,31] (see below). The structure of the *E. coli*-expressed gp20 protein [32] and of the same within the capsid vertex [33] have been determined through cryo-EM to near-atomic resolution. The 12 gp20 subunits form a flying-saucer-shape oligomer with a central channel (Figure 3A–C) that serves as a conduit for DNA packaging into the capsid during head assembly and as an exit during infection. The gp20 subunits have a fold similar to that of the portal proteins of other phages and herpesviruses [34,35,36], indicating their common evolutionary origin. 

The gp20 portal subunit can be subdivided into clip, stem, wing, and crown domains/regions (Figure 3D). The clip region is exposed outside the capsid shell and is involved in interactions with different proteins during virion assembly, namely the DNA-packaging motor protein, gp17, for genome packaging [31] and, later, the dodecameric neck protein, gp13, which through an interaction with the hexameric gp14 [37] seal the portal vertex after headful genome packaging. The assembled neck creates a binding site for the docking of the phage tail that is independently assembled. 

The gp20 clip region contains two positively charged residues near the channel entrance that might be involved in capturing the genomic DNA end at the initiation of the packaging process. The stem, wing, and crown regions are inside the capsid. In the full capsid, the crown and wing regions interact with genomic DNA, while the wing region also interacts with the major capsid protein capsomers surrounding the portal. The central stem domain containing two long antiparallel helices is negatively charged, while the small region in the clip domain near the channel entrance is positively charged. The modeling of a B-DNA helix into the portal channel shows that possible contacts between the portal protein and the DNA are confined to three polypeptide loops, separated from each other by approximately one helical turn of DNA [32]. The first “tunnel” loop connects the stem and the wing region, the second “channel” loop connects the clip domain with the stem helix α7, and the third “inner clip” loop is at the end of the clip domain (Figure 3D). Through conformational changes in the portal, some of these loops may constrict or expand the central channel. Thus, the portal may act a molecular valve that controls the flow of DNA into the capsid during packaging and out of the capsid during ejection [34].

Because the portal protein 12-mer is surrounded by the fivefold-symmetric gp23* capsid shell, there is a symmetry mismatch between the portal and the capsid, and each of the 12 portal subunits faces different regions of the gp23* shell. The structure of the symmetry-mismatched portal–capsid interface was resolved to near-atomic resolution in the asymmetric cryo-EM reconstruction, which included the portal protein and the five surrounding gp23* capsomers. The reconstruction showed the remarkable structural morphing of the portal to compensate for the symmetry mismatch [33]. Namely, the flexible components of the portal protein, in the periphery of its wing region, display significant conformational differences among the 12 portal subunits, whereas the gp23* shell surrounding the portal strictly obeys the fivefold symmetry and does not show any significant conformational changes induced by the portal [33]. The flexible portal components showing large structural variations include the N-terminal “whisker” Met1-Leu6, the “hairpin” Arg185-Glu204, and the “loop” Asp209-Lys227 (Figure 3E–G), all of which are parts of the portal wing. 

The cryo-EM reconstruction showed that, due to the the portal’s flexibility and structural adaptation, similar interactions between different portal subunits and the surrounding capsid protein molecules repeatedly occur [33]. For instance, the N-terminal whiskers of portal subunits 1 (*p*) and 6 (*p. + 5*) interact with the two fivefold-symmetry-related regions of the gp23* shell and form potential methionine-metal clusters with gp23* molecules (Figure 3H,I). Furthermore, similar salt bridges occur between the fivefold-symmetry-related regions of the gp23* shell and the portal subunits whose numbers have the *p*, *p. + 5*, and *p. + 7* relationship [33]. Owing to the 12-fold symmetry of the portal and the fivefold symmetry of the gp23* shell, portal subunits obeying this *p*, *p. + 5*, and *p. + 7* relationship encounter similar gp23* environments, which differ by either a +6° or a −6° rotation. The cryo-EM structure shows that the flexible components of the portal protein morph to compensate for these environmental differences and reach similar interaction partners [33].

The cryo-EM structure also showed that the portal and capsid axes are slightly misaligned, resulting in a 0.9° tilt of the portal with respect to the capsid [33]. This portal tilt results in favorable hydrophobic interactions of some portal subunits with the neighboring gp23* molecules. The portal tilt axis was found to be roughly parallel to the line connecting the two potential methionine-metal clusters formed by the N-terminal methionine of subunits 1 and 6 of the portal with two methionines and one histidine of the neighboring gp23* subunits, though the coordinating metal atom has not been identified. From the structural disposition, it appears that these clusters serve as anchors attaching the portal to the capsid and might be important for regulating the portal–capsid interactions during assembly, capsid expansion, and genome packaging. Consistently, genetic and biochemical studies showed that the length of the portal N-terminal whisker is critical for the phage viability. Shortening the six-amino-acid whisker by one or two amino acids did not affect phage viability, whereas three or four amino acid deletions resulted in lethality and failure to correctly assemble the capsids [33]. The shortened whisker presumably disrupted the potential methionine-metal clusters and was unable to properly interact with the capsid protein subunits.

### 2.3. Decoration Proteins

The T4 head has two decoration proteins, Hoc (highly immunogenic outer capsid protein) and Soc (small outer capsid protein), that bind to the capsid surface during the late stage of capsid assembly [16,38,39,40]. The wild-type prolate capsid has 155 binding sites for Hoc, one per gp23* capsomer, and 870 binding sites for Soc, one per gp23* subunit, except for the gp23* subunits interacting with the gp24* subunits at the pentameric vertices (Figure 1B–D). 

One Hoc subunit attaches to the center of each gp23* hexameric capsomer. The elongated fiber-like Hoc molecule consists of four domains, where the C-terminal domain is responsible for the capsid attachment [39,41]. The three N-terminal domains of Hoc exposed to the solvent have immunoglobulin (Ig)-like folds [39] (Figure 1C). Because Hoc monomers bind to the centers of gp23* hexamers, each Hoc molecule can randomly bind in one out of six possible orientations related by the hexamer axis. This leads to an enormous number of combinations of different orientations in which 155 Hoc molecules can bind to their sites on the capsid surface. This in turn leads to diverse Hoc orientation patterns exposed on different T4 particles. The Hoc protein is nonessential under laboratory conditions and has only a marginal effect on capsid stability. However, the Ig-like domains of Hoc probably help the phage to bind to different surfaces [39,42,43,44]. Biochemical experiments showed that the expressed Hoc protein can bind to the *E. coli* surface [39]. Therefore, Hoc may be beneficial to the phage in that it may help the virion to stay attached to the cell while the tail fibers search for their receptors. In addition, Hoc might allow the virions to attach to bacterial cells and use them as vehicles to travel to different locations [39]. Additionally, because *E. coli* and T4 populate the human gut, Hoc may help the phage to interact with molecules abundant on the surfaces of cells in the gut environment. A recent study found that a Hoc mutation (Asp246 to Asn) caused altered phage binding to fucosylated mucin glycans and provided the mutant phage a competitive fitness advantage over the wild-type phage in the gut-on-a-chip mucosal environment [45].

**Figure 4:**
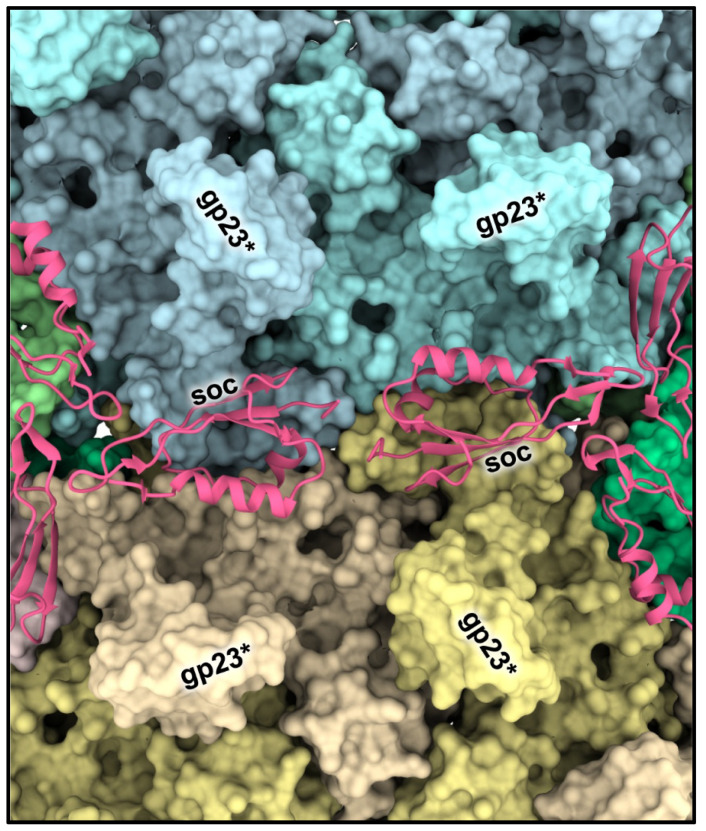
Soc molecules clamp the capsomers of the mature expanded T4 head. Each Soc subunit (shown as a ribbon diagram in magenta) interacts with two neighboring gp23* capsomers (shown as surfaces in various colors) reinforcing the capsid structure (PDB ID: 5VF3).

The tadpole-shape Soc molecules bind to the capsid surface at the interfaces between adjacent gp23* capsomers and clamp the capsomers [18,38] (Figure 4; Figure 1C). The Soc structure is different from the decoration proteins of other phages that bind to intercapsomer interfaces [46,47]. Although Soc protein is not essential for phage assembly, it reinforces the capsid shell and stabilizes it against extremes of pH (above pH 10.6) and temperature [38,40]. Interestingly, the high-resolution icosahedral structure of the empty isometric head [18] showed that the Soc binding sites are not equally occupied. In the isometric head, the occupancies varied from ~0.4 to ~0.6 and correlated with the size of the angle between the planes of adjacent hexameric capsomers clamped by Soc. The largest Soc occupancies and the largest intercapsomer angles were observed near capsid vertices where there is the largest deviation from a planar hexagonal array. Therefore, Soc molecules prefer to bind and reinforce the capsid near the vertices, where there is probably the greatest strain in the gp23* hexagonal lattice.

Both Hoc and Soc exhibit exquisite specificity to T4 capsid and nanomolar binding affinity. As these are nonessential for phage infection, Hoc and Soc have been extensively used as adapters to efficiently display pathogen epitopes, antigens, and large complexes at high density on the capsid surface. Lindsay’s laboratory and Rao’s laboratory, as well as other laboratories, have used this platform to design vaccines against a number of bacterial and viral diseases [9,48,49]. When administered to animals, the antigen-decorated phage nanoparticles induce robust and broad immune responses that include neutralizing antibodies, T cell responses, and mucosal responses. Immunized mice, rats, rabbits, and macaques were completely protected against challenges with lethal doses of infectious disease agents such as *Bacillus anthracis* (anthrax), *Yersinia pestis* (plague), SARS-CoV-2 (COVID-19), and H1N1 influenza A (flu) [50,51,52]. It appears that the surface architecture of the T4 phage capsid mimics the pathogen-associated molecular patterns (PAMPs), triggering strong immune responses by the host. In addition to the delivery of vaccines, Hoc and Soc have also been used to display targeting molecules that help deliver genes packaged in T4 heads to human cells, which in future could be developed as gene therapy devices to treat various genetic diseases [10].

## 3. Head Assembly

### 3.1. Assembly Pathway and Maturation

**Figure 5:**
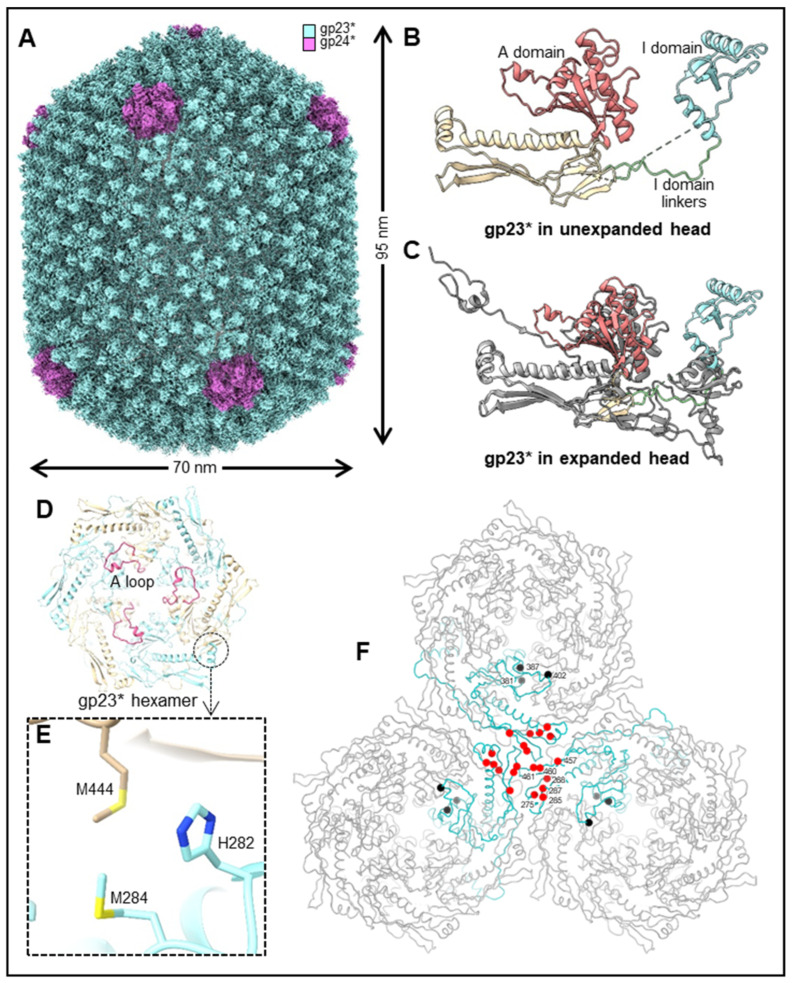
Structure of the phage T4 unexpanded head. (**A**) Cryo-EM structure of the phage T4 unexpanded head (EMD-32103). (**B**,**C**) Ribbon diagrams of gp23* in unexpanded (panel (**B**)) and expanded (panel (**C**)) conformations (PDB ID: 7VRT). (**D**,**E**) One portal vertex-surrounding capsomer (**D**) in the unexpanded head showing residues from two adjacent gp23* subunits forming a potential metal-binding cluster (**E**), whereas in the expanded head, the residues are from the same gp23*subunit (Figure 2). (**F**) Mutations involved in inter-capsomer interactions that alter the capsid length are clustered around the quasi-threefold axes (red dots), whereas the gp24 bypass mutations that do not alter capsid length affect intra-capsomer interactions (black dots).

Like other tailed dsDNA phages and herpesviruses, T4 assembles its capsid via the formation of a DNA-free proteinaceous precursor, called procapsid or prohead (Figure 5A) [4,16,53]. The prohead formation is initiated by the gp20 portal protein dodecamer, attached to the inner membrane of the *E. coli* cell. The assembly of the membrane-bound portal initiator depends on a viral chaperone, gp40, and it can be crosslinked to host membrane proteins Tig, DnaK, and YidC [54]. It appears likely that DnaK transports the protein to the membrane, while YidC may function as a membrane-associated chaperone allowing the binding of gp20 to the surface of the lipid bilayer until the prohead assembly has completed (Quinten and Kuhn, personal communication).

The portal protein nucleates the assembly of the inner scaffolding core and the gp23 capsid protein shell surrounding the core. The inner core is composed of the major core protein, gp22 (~580 copies); the prohead protease, gp21 (~55 copies); internal proteins IPI (~360 copies), IPII (~360 copies), and IPIII (~370 copies); gp67 (~340 copies); gp68 (~240 copies); and gpAlt (~40 copies) [16,17]. The IPI, IPII, IPIII, gp68, and gpAlt proteins are not essential for assembly. However, IPI is beneficial for the phage in that it is injected into the host, along with the T4 genome, and serves as an inhibitor of the GmrSD endonuclease, thus protecting the phage DNA from restriction [55,56]. The gpAlt protein ADP-ribosylates the host RNA polymerase and increases its affinity for the early T4 promoters, thus leading to the preferential transcription of the early T4 genes [57].

The mechanisms that localize certain proteins in the scaffolding core remain mysterious, and the structure of the core remains unresolved. Interactions between the core proteins lining the scaffold and the regions of the major capsid protein that line the capsid interior would be clearly essential for coassembling the capsid and the core. Lindsay and colleagues discovered that a ~10 aa N-terminal capsid-targeting sequence (CTS) is conserved in IPII and IPIII, but not in other core proteins [58]. This CTS sequence when attached to foreign proteins such as the green fluorescent protein (GFP) and expressed during T4 infection localized these proteins in the core structure [59]. Upon completion of the icosahedral prohead assembly, the gp21 protease becomes active and starts to digest the inner scaffolding core. The protease is pentameric, with a shape of a starfish, where the catalytic centers are in the starfish arms [60]. The X-ray structure of gp21 in the preactive form shows that the N-terminal region of the protein blocks its catalytic center, indicating that the activation mechanism involves the self-cleavage of nine N-terminal residues. Biochemical studies of T4 mutants [61] have suggested that the protease activation is triggered by the attachment of the gp24 protein to the prohead vertices. Together with the pentameric nature of gp21, these studies suggest that in the prohead core, the gp21 pentamers are initially located near the pentameric vertices. Later, during the core degradation, the protease can diffuse to the interior of the prohead and digest the core and capsid proteins. 

The active gp21 protease degrades gp22, gp67, and gp68 into small peptides and cleaves off small N-terminal peptides from the core proteins IPI, IPII, IPIII, and gpAlt [16,17]. The peptides presumably escape from the prohead through openings in the prohead shell, liberating space for packaging the phage genome. In addition, the protease removes the 65-residue N-terminal region of the major capsid protein gp23 and the 10-residue N-terminal peptide of the vertex protein gp24, producing gp23* and gp24*, respectively. The protease pentamers also degrade each other, apparently leaving about one pentamer in the capsid [17,60]. 

Following the maturation cleavages, the “empty” prohead is released from the cell membrane, and the clip domain of the portal becomes accessible for the assembly of the DNA-packaging motor protein as a pentamer. The pentameric packaging motor translocates genomic DNA into the capsid through the portal channel fueled by ATP hydrolysis [31]. During packaging, the capsid expands and increases its inner volume by an additional 70% [15]. The expansion is accompanied by profound conformational changes in the gp23* shell, and this expansion creates binding sites for Hoc and Soc. Genome packaging continues until the head becomes full (~1.02 to 1.03 of the unit-length T4 genome is packaged), and a signal is sent through the portal to the gp17 motor, triggering packaging termination. Next, gp17 cuts the packaged DNA from the rest of the DNA concatemer by using its nuclease activity and departs from the portal vertex [2,62]. The exposed portal clip domains now interact with 12 gp13 subunits, to which six gp14 subunits attach [37]. The (gp13)_12_-(gp14)_6_ complex seals the portal vertex of the DNA-full head and creates the binding site for the docking of the phage tail. 

### 3.2. Expansion

Although the T4 head has been studied for many decades, the structure of the unexpanded gp23* shell has remained unresolved because the prohead particles are fragile and can spontaneously expand in vitro and in vivo [1], with no DNA being packaged. The difficulty of producing large amounts of unexpanded prohead particles for structural study has recently been overcome by the pre-expression of the gp20 portal protein in *E. coli* cells and the infection of these cells with a T4 mutant deficient in the portal protein, the DNA-packaging motor, and the neck and tail assembly (*10am13am17am20am*) [15]. The empty proheads isolated from these infections did not expand, although their inner scaffolding core was completely degraded by the gp21 protease, and their gp23 and gp24 proteins underwent normal maturation cleavages. This suggests that the portal protein plays an important role in triggering the capsid expansion. Fang et al. [15] suggested that the pre-expressed portal protein is unable to trigger expansion because when the portal is expressed in the absence of its interacting partners (gp40, gp23, and scaffolding core proteins), it might assemble in a conformation that is different from that in a natural T4 infection. The 5.1 Å-resolution cryo-EM structure of the unexpanded prohead was determined (Figure 5A–C), and its comparison with the 3.4 Å-resolution structure of the mature capsid [15] revealed dramatic conformational changes during capsid expansion and stabilization. 

Overall, after the expansion, the capsid length increases from ~950 Å to ~1200 Å, and the width increases from ~700 Å to ~860 Å (Figure 5A), while the capsid wall becomes thinner. Consequently, the capsid volume increases by an additional ~70%, which is essential to accommodate the complete viral genome. In the unexpanded prohead, each hexameric gp23* capsomer adjacent to a pentameric vertex is skewed into two gp23* trimers, roughly related by a twofold axis. However, these gp23* capsomers become almost sixfold symmetric after expansion.

In the unexpanded prohead, adjacent capsomers are bound to each other mainly near the quasi-threefold axes of the capsid shell via hydrophobic interactions between the three adjacent P-loops and by electrostatic interactions between the short, negatively charged α-helix and a positively charged β-strand region from adjacent P domains. These interactions are largely preserved after the capsid expansion, indicating their importance for maintaining capsid integrity. Moreover, the structures of these regions near the quasi-threefold axes also remain relatively unchanged, indicating that they act as anchor points around which the capsid subunits rotate and twist during the expansion process. 

During expansion, the disordered N-terminal regions of gp23* and gp24* migrate from the prohead interior to the outer capsid surface and form ordered structures, N-arms, and N-fists (in case of gp23*), which form extensive interactions with the neighboring subunits, stabilizing the expanded shell. Furthermore, the gp23* protein has an unusual A-loop (Figure 5D), which plays a critical role in A domain–A domain interactions and the stabilization of hexameric capsomers in the unexpanded prohead. However, in the expanded structure, this loop folds back to the bottom of its own A domain and makes much fewer intracapsomer contacts.

In both the unexpanded and expanded capsids, the I domain of gp23* sits on top of an adjacent subunit from the same capsomer. However, the binding interfaces between the I domain and the adjacent subunit are very different in the unexpanded and expanded structures. After expansion, the interactions between the I domain and the adjacent subunit become more extensive, and the binding interface area increases from ~460 Å^2^ to ~650 Å^2^. The I domain linkers, connecting the I domain to the rest on the structure, are partially disordered in the unexpanded structure but become ordered and form intercapsomer interactions stabilizing the capsid shell. In addition, these linkers, together with N-fists, create binding sites for the Soc protein, which further reinforces the expanded capsid. All these conformational changes occurring during expansion result in a substantial increase in capsid stability, which is necessary to endure the pressure imposed by the tightly packed DNA.

Capsid expansion is probably triggered at the portal vertex and then propagates as a “wave” through the entire capsid structure [15]. The expanded head structure shows that the N-terminal methionines of portal subunits 1 and 6 coordinate with Met98, Met284, and His282 from adjacent gp23* molecules to form potential metal-binding clusters (Figure 5E). These clusters probably anchor the portal to the capsid shell. An analysis of the unexpanded shell structure suggests that these clusters are likely also present in the unexpanded prohead, although the portal appears to be in a more dynamic state. Furthermore, the composition of the gp23* molecules that form the clusters is different. The Met98 residue of gp23* in the cluster is replaced by Met444, belonging to a neighboring gp23* subunit (Figure 5E), rather than that of the same subunit in the expanded head (Figure 3H,I). This represents an expansion-associated conformational switch in the capsid subunits at the portal–capsid interface, which probably acts as a trigger for expansion [15]. 

Additional observations from independent studies are consistent with the portal as the epicenter of expansion: (i) capsids assembled using a pre-expressed portal protein did not spontaneously expand, contrary to the capsids produced during phage infection [15], (ii) mutant heads in which some of the portal protein subunits were replaced by a recombinantly expressed portal–GFP fusion were found to remain in the unexpanded conformation [63], (iii) electron micrographs of giant heads showed some particles in which expansion was interrupted halfway, where the expansion front was perpendicular to the capsid axes [64,65]. These points lead to the conclusion that expansion is polar and propagates as a wave from the portal vertex.

How does the conformational switch occur at the portal–capsid interface? As the genome becomes encapsidated, the internal pressure exerted by the packaged DNA would push down on the portal dodecamer. There is evidence that the first packaged DNA end may be clamped at the portal vertex [66,67]. Given that the portal is anchored to the capsid via the metal-binding clusters, this would cause a pull on gp23* molecules, inducing conformational changes. Consequently, the remodeling of the metal-binding clusters would occur, leading to unexpanded-to-expanded transitions in gp23* subunits anchored to the portal. These conformational transitions would then trigger energetically favorable conformational changes in their neighbors, causing a “domino effect”. Thus, the expansion wave initiated at the symmetry-mismatched portal–capsid interface would propagate through the portal-proximal icosahedral cap and then through the capsid midsection to the distal cap [15].

### 3.3. Length Control

The wild-type T4 head has an elongated midsection, characterized by the triangulation number T_mid_ = 20, the length of which is strictly controlled during assembly. However, several single-point mutations that alter the capsid length and result in mixtures of isometric (icosahedral), intermediate, prolate, and giant heads were found in gene 23 [68,69,70] (Figure 5F). For example, a single Ala275-to-Thr substitution results in the production of ~80% of the isometric heads, together with ~20% of the wild-type and intermediate-length heads. This mutation maps to the short negatively charged helix of the P domain involved in electrostatic interactions with the adjacent capsomer. A mutant phage containing this mutation was used to produce isometric particles for the high-resolution icosahedral cryo-EM reconstruction of the capsid [18].

Generally, the length-changing mutations cluster near the quasi-threefold axes that relate adjacent hexameric capsomers [15,18] (Figure 5F). These mutations probably affect the intercapsomer interactions and modulate angles between adjacent gp23 capsomers, which, in turn, result in an altered capsid length. An analysis of the intercapsomer angles in the prohead [15] shows that small angles (2–5°) occur more frequently in the midsection than in the caps. Mutations affecting intercapsomer interactions can make certain intercapsomer angles more or less favorable. For example, if a mutation makes small intercapsomer angles more favorable, the midsection would further elongate during assembly, generating “giant” heads. If, on the other hand, a mutation makes small angles less favorable, the gp23 capsomers would assemble into isometric heads (having shorter midsection). Thus, small changes in the intercapsomer interactions may lead to profound shifts in viral capsid morphology and volume. Because a single amino acid substitution can switch the capsid morphology from prolate to isometric (and vice versa), it is tempting to speculate [15] that T4 phage originally might have had an icosahedral capsid, but a small change in the intercapsomer contacts changed it to a prolate capsid that could package ~50 kbp more DNA, providing space for extra genes that conferred survival advantages.

## 4. Genome Packaging

Genome packaging in phages and viruses is carried out by a powerful packaging machine that generates up to 80–100 pN of force [30,71,72,73,74,75]. Such high forces are essential to compact the dsDNA inside the capsid to near-crystalline density as an ordered condensate, against forces that oppose order, bending, and electrostatic repulsion. The T4 packaging machine consists of three key components: a portal (gp20), a motor (gp17), and a regulator (gp16) [3,4]. The motor, an oligomer ring formed by gp17, is central to the energetic translocation mechanism, while the gp20 portal and the gp16 regulator play supporting, yet critical, roles in the packaging mechanism and in generating the fully packaged head. 

The gp17 motor protein and the gp16 regulator protein are referred to as “terminases” (TerL for gp17 or large terminase and TerS for gp16 or small terminase) because these proteins, in addition to their roles in packaging, form a hetero-oligomeric complex that makes cuts in the concatemeric phage genome to generate the termini [5,76,77]. After the first cut, the terminase-DNA complex docks on the portal, presumably orienting one of the cleaved ends into the portal channel and initiating DNA translocation into the capsid. The T4 TerS-TerL terminase complex is unstable in vitro [2], and the structure of the complex is unknown. The dynamic interactions and the remodeling of the complex during genome cleavage and genome translocation are also poorly understood. However, the structural and functional aspects of individual TerS and TerL proteins have been well characterized.

### 4.1. TerS

**Figure 6:**
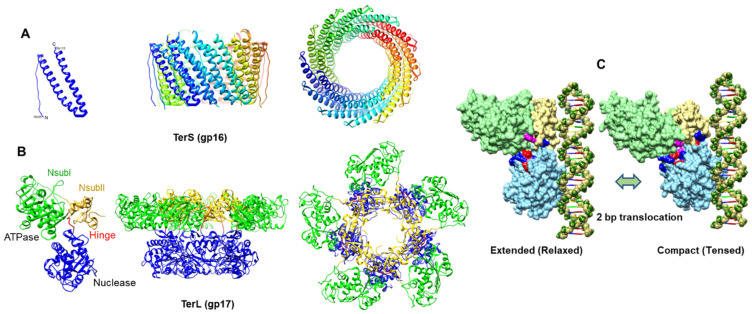
Structures of the phage T4 TerS and TerL terminase/packaging proteins. (**A**) Structures of gp16 TerS oligomerization domain from the T4-related phage 44rr2. A gp16 monomer and the side and top views of the 12-mer are shown (PDB ID: 3TXS). (**B**) Structural models of full-length gp17 TerL; monomer (PDB ID: 3CPE) and side and top views of pentameric motor (PDB ID: 3EZK) derived from the cryo-EM density. (**C**) Structural models of extended (relaxed) and compact (tensed) conformational states of TerL, showing ion pairs at the interface of ATPase and nuclease domains. Acidic residues are shown in red spheres, basic residues in blue spheres, and hydrophobic/uncharged residues in purple spheres.

The 18 kDa small terminase, TerS. is dispensable in vitro but essential in vivo for DNA packaging [2,78] (Figure 6A). Chain-terminating mutations in gene 16 lead to lethality and accumulate mostly empty proheads and some partially filled heads. Previous mutational analyses suggested that gp16 might be important for viral genome recognition and packaging initiation [79,80], whereas biochemical studies suggest that gp16 acts as a regulator of gp17 and motor functions [81,82]. 

The overall structure of phage T4 TerS and other small terminases is conserved. It consists of three domains: a central oligomerization domain that forms the core, an N-terminal domain containing a helix-turn-helix motif involved in potential DNA binding, and a C-terminal domain that might be involved in gp17-ATPase stimulation [82]. The latter also appears to determine the specificity of the interaction with gp17. Swapping this region between phages T4 and RB49 switches the ATPase stimulation specificity from T4 gp17 to RB49 gp17 and vice versa [81,83].

The electron microscopy of purified gp16 shows oligomers of single rings and side-by-side double rings [79,83], and mass spectrometry revealed that the single and double rings correspond to 11-mers and 22-mers, respectively [84]. Sequence analyses predicted coiled–coil motifs in gp16 and other phage TerS sequences, consistent with their propensity to form stable oligomers [85]. Oligomerization occurs through these coiled–coil interactions between neighboring subunits. Mutations that perturb these interactions cause defects in oligomerization [85]. 

The X-ray structure of the central oligomerization domain of the T4-related phage 44rr2 TerS has been determined [86] (Figure 6A). Consistent with the mass spectrometry data, the structure showed 11-mers and 12-mers that were stabilized by extensive hydrophobic and electrostatic interactions between the two long helices of the central domain, as predicted by biochemical analyses. These helices form a tightly packed interface generating a cone-like structure that has a height of about 40 Å and an inner diameter of 32 Å (11-mer) to 37 Å (12-mer) at the wider end and 24 Å (11-mer) to 27 Å (12-mer) at the narrower end. 

The function of the oligomerization domain appears to be to display the N-terminal DNA-binding helix-turn-helix domains around the ring structure [87]. Such an arrangement would allow for the wrapping of phage genomic DNA around the TerS ring. An interaction with gp17 through its C-terminal domain would lead to the formation of TerS-TerL complex that then proceeds with DNA cleavage and insertion of DNA end into the portal channel to initiate packaging. Though models have been proposed in which DNA passes through the TerS channel and may even be threaded during DNA translocation, the evidence is not consistent with such models [88,89]. Mutations of residues that line the channel, including partial or complete deletion of one of the channel helices, do not lead to loss of DNA binding in vitro or cause lethality in vivo. 

A favorite model of Lindsay, referred to as the “synapsis” model, was based on a large amount of genetic data from his laboratory, beginning with the thesis work of his graduate student Du Gong Wu [90], that implicated a link between recombination and packaging initiation. At the center of the synapsis model is the oligomeric gp16, as double or multiple rings that recognize putative pac sites in the viral genome. Interactions between the rings and the pac sites would generate a higher order TerS-genome complex in which the concatemeric viral genome is aligned as a “bundle” of unit-length genomes, similar to chromosome segregation in higher organisms [91]. This structure would then be resolved into individual genomes by packaging into capsids. However, there are no defined pac sites demonstrated in T4 genome, as was documented in the classic pac phages such as SPP1 and P22. Lindsay’s work identified putative pac sites in g16 and g19 on the basis of evidence that these sites enhanced site-specific recombination in a gp16-dependent manner [92]. The synoptic complex is thought to facilitate interactions with TerL, and the TerS-TreL-DNA ternary holoterminase complex thus formed cuts DNA and attaches the end to the prohead to initiate the processive packaging of the concatemeric genome. In this model, the rings are predicted to be helical lock washers rather than flat, closed ring structures [93]. Such complexes might be difficult to crystallize, but cryo-EM might be able to generate the structures in the future, which would fill an important gap in understanding the mechanism of the initiation of phage genome packaging. 

Another key function of gp16, discovered rather unexpectedly, was that it stimulates the gp17-ATPase activity by >50-fold [78]. Such a stimulation was also observed in many other phages, in subsequent studies [94,95]. Hence, it is a common function of small terminases, probably linked to ATP-powered translocation. This is also consistent with the gp16 stimulation of in vitro DNA-packaging using crude mutant-infected extracts that also contain the DNA replication/transcription/recombination proteins. This system, in many respects, mimics the in vivo packaging of viral genomes [2,78]. However, the TerS gp16 is not required in a defined in vitro packaging assay consisting of only two purified components; proheads and gp17. On the other hand, gp16 inhibits packaging in this defined system [96,97]. 

Gp16 stimulates gp17-nuclease activity in vivo, and it does so in vitro in the presence of ATP, but it inhibits nuclease when ATP is absent [62,81]. Furthermore, gp16 inhibits the binding of gp17 N-terminal domain to DNA [98]. Both the N- and C-domains of gp16 are required for ATPase stimulation and nuclease inhibition, and the maximum activity was observed at a ratio of one gp16 oligomer to one gp17 [81]. These results are compelling, implicating communication between the gp17 ATPase and nuclease domains, which is modulated by the nucleotide-binding state and the interaction with gp16 TerS. A communication track through which signals might be transmitted has been proposed based on structure and molecular dynamics simulations [62]. 

The gp16 TerS also contains an ATP binding site with broad nucleotide specificity [79,81], but it lacks the canonical ATP binding signatures such as Walker A and Walker B [83]. However, curiously, nucleotide binding was not correlated with gp17-ATPase stimulation or gp17-nuclease inhibition. Therefore, the exact function(s) of gp16′s ATP binding is unknown. 

Taken together, the above observations suggest that gp16 regulates the DNA-packaging machine and its components. Although its primary function might be the recognition of the viral genome, it also modulates the ATPase, nuclease, and translocase activities of gp17 for efficient packaging initiation and DNA translocation. The overall model, therefore, is that a packaging initiation complex consisting of gp16, gp17, and DNA, forms potentially at preferred sites (putative pac sites) on the concatemeric viral genome. Gp17 then makes a cut and inserts one of the ends into the portal channel while itself assembling as an oligomeric motor and together forming the packaging machine (complex of motor, portal, and DNA). The gp17 ATPase of the motor is then stimulated by gp16, causing the firing of the ATPases in rapid succession, “jump-starting” the DNA-packaging machine (cranking the engine) [86,99].

### 4.2. TerL

The 70 kDa TerL, the large terminase subunit, is the motor protein of the T4 DNA-packaging machine [2] (Figure 6B). TerL consists of two domains: an N-terminal ATPase domain and a C-terminal nuclease domain. The N-terminal domain encodes the canonical ATPase signatures, including the Walker A and Walker B motifs, and the catalytic carboxylate [83]. The C-terminal domain contains two potential DNA-binding grooves and a nuclease active formed by a catalytic metal cluster containing conserved aspartic and glutamic acid residues coordinating with Mg [100].

#### 4.2.1. ATPase

Extensive biochemical studies have established that gp17 alone, in the absence of gp16, is sufficient to efficiently package DNA in vitro [2]. However, its ATPase activity in the absence of DNA packaging is weak (K_cat_ = ~1–2 ATPs hydrolyzed per gp17 molecule/min). The small terminase gp16 stimulate the gp17 ATPase, by as much as 50–100 fold [78,95]. Although gp16 stimulation might be essential for packaging initiation in vivo, it is not so for the same in vitro in the presence of excess gp17. Furthermore, during active packaging, conformational transitions in the packaging machine stimulate the gp17 ATPases to fire in a continuous burst (see below). 

Bioinformatic analyses predicted the catalytic residues of the ATPase center in the N-terminal domain of gp17 and other large terminases [83]. Extensive molecular genetics and biochemical analyses demonstrated that these predicted catalytic residues are essential for the ATPase and DNA-packaging activities [101]. Even highly conservative substitutions in these catalytic signatures, such as changing an aspartic acid to glutamic acid and vice versa, resulted in the complete loss of DNA packaging. These data for the first time provided compelling evidence that this ATPase center provides energy for powering DNA translocation [102,103]. 

One of the ATPase mutants in which the Asp-Glu residues corresponding to the Walker B and catalytic carboxylate motifs were switched to Glu-Asp showed tighter binding to ATP, although the mutant protein failed to hydrolyze ATP [102]. Remarkably, the mutant ATPase domain readily formed crystals in ATP-bound, ADP-bound, and apo (nucleotide-free) states and the atomic structures were determined up to ~1.8 Å resolution [104]. The ATPase domain is a relatively flat structure with two subdomains (Figure 6B): a large subdomain I (NsubI) with the classic nucleotide-binding Rossmann fold and a smaller subdomain II (NsubII) forming a cleft in which ATP binds. All the previously predicted catalytic residues by biochemical and genetic analyses were found to be in the ATP binding pocket, forming a network of interactions with bound ATP. The catalytic pocket also contained a cis-arginine finger, which was also predicted by genetic analyses and was proposed to trigger βγ-phosphoanhydride bond cleavage. Additionally, the structure showed an adenine binding motif, an ATPase coupling motif (Motif III), and a loop near the adenine binding motif that exhibited significant movement in response to ATP hydrolysis. It is reasonable to consider that all of these components might be directly involved in the ATP energy transduction mechanism.

#### 4.2.2. Nuclease

Early studies established that when gp17 is overexpressed in *E. coli*, it exhibited sequence-nonspecific endonuclease activity, similar to that found associated with the purified gp17 in vitro, apparently producing blunt ends [105,106]. Similar activities have since been demonstrated in many of the well-characterized pac phage TerL homologs [107,108,109,110]. Biochemical and structural studies suggest that this activity probably makes packaging initiation and headful termination cuts in vivo during phage infection.

A histidine-rich site was identified in the C-terminal domain of gp17 by random mutagenesis and selection of mutants that are deficient in nuclease activity [111]. Sequence alignments and extensive site-directed mutagenesis of this region mapped a cluster of aspartic acid and glutamic acid residues that are conserved in all phage TerLs, and they are essential for DNA cleavage, as determined by the nuclease assays [112]. In contrast to the ATPase mutants that lost the gp16-stimulated ATPase activity, these mutants retained the ATPase activity but lost the DNA cleavage activity. The mutants could, however, package DNA in vitro if the substrate is an already-cut linear DNA, but they failed to package circular DNA because it required cutting to generate an end. 

The X-ray structures of the C-terminal nuclease domain of T4 gp17 and its homolog from the T4-family phage RB49, which has ~72% sequence identity, have been determined [31,113] (Figure 6B). The nuclease domain, unlike the ATPase domain, has a more globular structure with an RNAse H fold containing antiparallel β-strands, similar to that found in resolvases, RNase Hs, and integrases. Importantly, the structure showed all the predicted catalytic residues: Asp401, Glu458, and Asp542 forming a catalytic triad and coordinating with a Mg ion. In addition, the structure showed a putative DNA-binding groove lined with a number of basic residues where the acidic catalytic metal center was buried at one end of this groove. Together, these constitute the nuclease cleavage center of the gp17 large terminase.

#### 4.2.3. Translocase

DNA translocation requires both the ATPase and nuclease domains as part of the full-length gp17. Again, the Glu-Asp mutant (ED mutant) of the full-length gp17 readily crystallized, and the structure was determined to 2.8 Å resolution (Figure 6B) [31]. No significant conformational changes were observed in the N- and C-domain structures of the full-length gp17 when these were superimposed with the individually crystallized domains, as described above. However, the full-length TerL structure exhibited unique features. It contains a flexible “hinge” or “linker” domain that connects the ATPase and nuclease domains, which is essential for DNA packaging. In the absence of this hinge, the individual ATPase and nuclease domains retained the respective functions but completely lost the DNA translocation activity [100]. 

Additionally, the N- and C-domains share a >1000 square Å complementary surface area consisting of five charged pairs and hydrophobic patches at the interface [31] (Figure 6C). There is also a bound phosphate ion associated with the C-domain in the crystal structure. When a B-form DNA was docked with one of its phosphates superimposed on the bound phosphate, guided by shape and charge complementarity, the DNA aligned with a number of basic residues, forming what appeared to be a shallow DNA groove. This groove was predicted to be involved in DNA translocation. Thus, it appears that the C-domain of phage T4 TerL has two DNA grooves on different faces of the structure, one that aligns with the nuclease catalytic site that is involved in DNA cutting and another that aligns with the DNA in the motor channel that is involved in DNA translocation.

The structural features of TerL suggest that gp17’s nuclease might be tightly controlled by ATP, as was observed in in vitro studies. The nuclease is active at the initiation and termination steps of DNA packaging, but not during translocation, when the nuclease groove would not be in contact with the DNA. This suggests that there is a mechanism in which the headful nuclease is regulated by a “communication track” that consists of residues from subdomain II, hinge, and a β-hairpin that relay conformational signals between the ATPase and the nuclease centers [62]. When the DNA is being actively translocated, gp17 would be in the nuclease-inactive state, and furthermore, its oligomeric motor structure (see below) would not allow the formation of an antiparallel dimer that is essential to simultaneously make the cuts in both the DNA strands.

### 4.3. Packaging Motor

A functional packaging motor complex is assembled at the portal vertex by simply mixing purified proheads and gp17. This complex, in a bulk in vitro assay, can package the 171 kb phage T4 DNA, or any linear DNA [96,97]. If short DNA molecules are added as the DNA substrate, the motor packages multiple molecules in succession, one molecule after another [114]. This can lead to head filling when large plasmid DNA molecules are used (~30 Kb), but with shorter DNAs, mostly partially filled heads are produced [99,115]. Although the unexpanded prohead is the likely precursor for DNA packaging in vivo, the expanded prohead, the partially-full head, the once-filled and emptied head, or even the nearly full head can efficiently package DNA in the in vitro assay. In fact, surprisingly, even the virion’s packaged DNA can be emptied, and the emptied phage head can be repackaged with DNA again and again [1,116,117]. 

Lindsay and his collaborators developed a novel fluorescence correlation spectroscopy assay [115] to analyze packaging in real time at the single-molecule level. The kinetics of the translocation of a 100 bp DNA labeled with rhodamine (R6G) was analyzed by determining the decrease in the diffusion coefficient of the label as the DNA is translocated and confined inside the 120 × 86 nm capsid. Furthermore, fluorescence resonance energy transfer (FRET) occurred between the DNA label and the packaged GFP molecules, which further confirmed the ATP-powered DNA translocation and packaging of multiple DNA molecules into the same capsid [115]. Additionally, these studies determined that the ends of the translocated DNA were 8–9 nm apart, suggesting that the DNA might be translocated as a loop, with one end likely fixed near the portal [66].

The above points are consistent with the measurements made using the dual optical tweezers system in which the packaging motor complexes, where gp17 is assembled on the prohead portal, are physically tethered to a microsphere coated with capsid protein antibody and the biotinylated DNA is tethered to another microsphere coated with streptavidin [118]. When the microspheres are brought together into near contact, the motor captures the DNA end and begins translocation in the presence of ATP. This system allowed for the recording of single packaging events in real time and the detailed analysis of the dynamics of the T4 packaging process [118]. These data showed that the T4 motor generates forces of at least ~60 pN, a power density of ~5000 kW/m^3^, and a rate as high as >2000 bp/s, among the highest recorded to date. The T4 motor does slip and pause during translocation, but these are relatively short and infrequent. When these do occur, the motor recovers and recaptures DNA, continuing translocation. The high rate of translocation observed with the phage T4 motor is consistent with the need to package its 171 kb size viral genome in about 5 min. The power exerted by the T4 motor is extraordinary, as evident when external loads were applied. At an external load of 40 pN, the T4 motor translocates at a remarkable speed of ~380 bp/s, and at a load of 60 pN, the motor can still translocate at a rate of ~100 bp/s. When scaled up to a macromotor, the T4 motor is approximately twice as powerful as a typical automobile engine. 

The speed of the packaging motor, and its mechanism and control, are of significant interest because it is tied to genome length and viral fitness. Phages such as T4 that package a large genome must compete with phages with smaller genomes by being able to package the genome in approximately the same amount of time. Hence, T4 would need a faster motor. Molecular genetic studies provided evidence that the T4 ATPase center encodes a speed controller consisting of appropriately placed hydrophobic residues in the microenvironment of the catalytic glutamate [119]. It appears that by controlling the rate of activation of the “lytic water” molecule by the catalytic glutamate, the rate of ATP hydrolysis and the motor speed are controlled. Mutations that reduced the hydrophobicity or introduced polar functional groups into this microenvironment resulted in a reduced packaging rate or even a loss of function. These mutants also show frequent pausing and “unpackaging”, a phenomenon involving the release of packaged DNA when the ATP concentration is limiting. 

The T4 motor, when ATP is limited, instead of having greatly reduced packaging rate, as was observed in the phi29 motor [120,121], it frequently pauses, causing unpackaging [122]. This does reduce the average speed of the motor but only slightly. Apparently, when the motor encounters an apo subunit (lacking bound ATP), which frequently happens under limiting ATP concentrations, it pauses, which results in the loosening of its grip on DNA. The DNA–motor interactions become misaligned and the packaged DNA is slowly released. When the subunit recaptures ATP, the motor subunits adjust and realign with the DNA, forming a tight grip, and translocation is restored. These results are consistent with the tight DNA gripping observed when the motor is bound to ATP analogs, but weaker gripping is observed when bound to ADP or AMP, and essentially no gripping in the apo state [117].

The cryo-EM structure of the T4 packaging machine is consistent with the single-molecule studies (Figure 6B). It shows five molecules of gp17 bound to the clip domains of the dodecameric portal vertex. The pentamer stoichiometry of the motor creates a second symmetry mismatch at the portal vertex. Two rings of density are seen associated with the portal [31], with a flat upper ring resembling the shape of the ATPase domain and a lower ring of globular domains resembling the nuclease domain. The portal-interacting residues appear to reside in a helix-loop-helix region that is associated with the hinge domain. A peptide corresponding to the helix-loop-helix sequence inhibits in vitro DNA packaging [123]. The portal and the motor form a channel, and the putative translocation groove in the C-domain faces the channel. The X-ray structures of ATPase and nuclease domains fit into the cryo-EM density. However, the resolution of the cryo-EM structure is low, ~30 Å, which is not sufficient to resolve the structural details. Attempts thus far to improve the resolution have not been successful, in part owing to the symmetry mismatch of portal–motor interactions and the inherently dynamic nature of the motor subunits. 

The pentamer stoichiometry of the T4 packaging motor has been confirmed by single-molecule fluorescence studies by using total internal reflection fluorescence microscopy (TIRF) [124]. By assembling packaging machines using Cy-3-labeled gp17, the number of subunits in individual machines that actively translocated Cy-5 DNA oligonucleotides have been counted. These measurements gave a value of five subunits per packaging motor. However, the orientation of the motor with respect to the portal has not been verified. Lindsay’s studies using FRET measurements of packaging machines containing GFP-labeled portal protein and red-fluorescent ReAsH-labeled gp17 showed that the C-terminus of gp17 is closer to the portal than the N-terminus [125]. This predicted an orientation opposite to that predicted by the cryo-EM structure. A high-resolution structure of the packaging machine is therefore desperately needed to resolve the orientation as well as to define the interactions between the portal and the motor.

### 4.4. Packaging Dynamics and Mechanism

#### 4.4.1. Electrostatic Force Generation

Of several initial models proposed to explain the mechanism of viral DNA translocation, the portal rotation model [126] has gained the most attention. In this model, portal and DNA act analogous to a nut and bolt, respectively. The unique symmetry-mismatched portal vertex consisting of the fivefold-symmetric capsid and 12-fold-symmetric portal creates asymmetric, flexible interactions between these two structures. These enable the directional rotation of the portal (nut), powered by ATP hydrolysis, causing the translocation of the DNA (bolt) into the capsid. However, the first X-ray structures of portals determined from phages phi29 and SPP1 did not reveal such a nut-bolt type architecture, although the structures are thought to be basically consistent with the portal rotation model. Thus, newer and more-detailed rotation-incorporating models such as the rotation-compression-relaxation model [127], the electrostatic gripping model [128], and the molecular lever model [129] were proposed. 

To directly test these models, Lindsay’s laboratory constructed GFP fusions to either the N- or C-terminal end of the T4 portal protein and demonstrated that up to ~one-half of the dodecamer positions can be occupied with the fusion proteins without any loss of prohead function. As compared to the wild-type, portals containing C-terminal GFP fusions but not N-terminal GFP fusions [125] lock the proheads in an unexpanded conformation unless the terminase packages DNA, suggesting that the portal plays a key role in controlling prohead expansion. This has been confirmed by recent studies that showed that the assembly of the portal dodecamer in the absence of other head assembly components locks the portal in a different conformation that stabilizes the unexpanded state of the head [15]. Fusion to GFP is not required. Expansion, however, is required to protect the packaged DNA from DNAse because the unexpanded heads are leaky, as demonstrated by FCS [63]. Importantly, the retention of DNA packaging by the GFP-modified portals is inconsistent with the portal rotation model in that rotation would require that the bulky C-terminal GFP fusion proteins rotate without encountering any clashes. 

Lindsay has also designed a more direct test by tethering the portal to the capsid through Hoc interactions [130]. As described above, Hoc binding sites appear in the expanded heads following capsid expansion. By taking advantage of this feature, unexpanded Hoc-minus proheads were prepared by replacing some of the portal subunits with N-terminal Hoc-portal fusion proteins. The proheads were then expanded in vitro at a low salt concentration to expose the Hoc binding sites, allowing the portal-fused Hoc to bind to the center of the nearest hexon. This would lead to tethering of one to five portal subunits to the capsid through Hoc bridges, as indicated by the protection of Hoc from proteolysis. These head particles are able to package DNA in vitro. Thus, both the genetic and biochemical approaches strongly suggested that the portal rotation could not be the mechanism for packaging [130]. This conclusion was further supported by single-molecule fluorescence measurements in Bustamante’s laboratory, which showed with 99% certainty that the phage phi29 portal subunits failed to show rotation [131]. Lindsay’s approaches and experimental designs were therefore critical to finally put the portal rotation to rest that narrowed down the plausible packaging models.

A second class of packaging models was proposed, in which the terminase not only provides the energy but also serves as a molecular motor that couples the ATP energy to the active translocation of DNA. In a specific model [132], ATP-hydrolysis-driven conformational changes in the terminase domains cause changes in the DNA-binding affinities of the motor subunits, resulting in the binding and releasing of DNA. These would lead to the inchworm-type linear translocation of DNA, reminiscent of the mechanisms proposed for helicases. The sequence alignments of gp17 and numerous large terminases identified an ATPase coupling motif (also known as Motif III) that is commonly present in helicases and translocases [132]. Mutations in the coupling motif lead to binding and hydrolysis of one ATP, but the ATPase does not turn over in a catalytic manner, resulting in the loss of both ATPase and DNA-packaging activities.

The cryo-EM and X-ray structures (Figure 6) are consistent with this model and further refine it by postulating a more detailed, structure-based, electrostatic-force-driven packaging mechanism [31]. The pentameric T4 packaging machine can be considered analogous to an automobile with a five-cylinder engine containing the following components: an “engine”, or the ATPase center in NsubI; a “wheel”, or the C-domain translocation groove that moves DNA; a “transmission” NsubII domain that couples the engine to the wheel via a flexible hinge; an arginine finger “spark plug” that fires the ATPase; and an “alternator”, charged pairs that generate electrostatic force by alternating between relaxed and tensed states that is then converted to mechanical movement of DNA (Figure 6C). The nuclease groove faces away from the translocating DNA and is activated when packaging is completed.

In the cryo-EM structure, the two lobes (domains) of the motor are separated (“relaxed” or “extended” state), whereas in the X-ray structure, the domains are in close contact (“tensed” or “compact” state) [31] (Figure 6C). In the compact state, the NsubII of ATPase is rotated by 6° degrees and the C-domain is pulled upwards by 7 Å, equivalent to 2 bp. The arginine finger between NsubI and NsubII is positioned toward the βγ phosphates of the modeled ATP, and the ion pairs are aligned.

In the extended conformational state (cryo-EM structure), the hinge is extended. The binding of DNA to the translocation groove and of ATP to NsubI locks the motor in translocation mode and brings the arginine finger into position, firing ATP hydrolysis. The repulsion between the negatively charged ADP(3-) and Pi(3-) drive them apart, causing NsubII to rotate by 6° degrees, aligning the charge pairs and the complimentary surfaces between the N- and C-domains. This generates electrostatic force, attracting the C-domain-DNA complex and causing ~7 Å upward movement resulting in the compact conformational state (X-ray structure). Thus, ~2 bp of DNA are translocated into the capsid in one cycle (Figure 6C). Product release and the loss of six negative charges causes NsubII to rotate back to the original position, misaligning the ion pairs and returning the C-domain to the relaxed state. 

The translocation of 2 bp would bring the translocation groove of the adjacent subunit into alignment with the backbone phosphates. DNA is then handed over to the next subunit by the matching motor and DNA symmetries. Thus, ATPase catalysis induces conformational changes, which generate electrostatic force, causing the directional motion of DNA into capsid. The pentameric motor translocates 10 bp (one turn of the helix) when all five gp17 subunits fire in succession.

There is evidence for this electrostatic-force-driven translocation mechanism. Single-molecule optical tweezer measurements have shown that mutations in the charged pairs at the N- and C-domain interacting surfaces result in an impairment of force generation, a reduction in motor velocity, and an increased frequency of pausing and slipping [133,134]. For instance, when the charge of one of the pairs was reversed, the motor velocity dropped to zero when a 60 pN external force was applied, whereas the wild-type motor still packaged at a rate of ~100 bp/s. Furthermore, molecular dynamics simulations have shown that the measured impairments correlated with the free-energy differences computed between the extended and compact conformational states, according to the changes made to the ion pairs at the interface. 

#### 4.4.2. DNA Structural Transitions

While Lindsay was in favor of this model and while his results were in agreement with it, he believed that the DNA was not translocated by a simple linear motion. His experiments with modified DNA substrates indicated a torsion-compression mechanism in which the portal grips the DNA while a power stroke is applied by the above conformational changes in the large terminase motor [135]. The DNA structure becomes compressed in the translocation channel between the portal and the ATPase motor, and releasing the grip would lead to DNA movement into prohead. The presence of nicks in <200 bp DNA substrates blocked translocation, suggesting that the energy stored as DNA compression may be dissipated by a nick. On the other hand, the apparently normal translocation of longer DNAs containing nicks or other abnormal structures by T4 and phi29 motors [136] is not consistent with this model. 

The use of a several kilobases-long linear DNA connected to a 90 bp Y-DNA structure showed packaging from the linear end of the molecule, but stalling occurred when the motor reached the Y-junction, as evident by the FRET signal between the dye-labeled Y-junction and the GFP-labeled portal [137]. Similar Y-DNA substrates containing FRET-pair dyes in the Y-stem separated by 10 or 14 bp gave a FRET signal that measured a distance reduction between the pairs of ~22–24%. These data also suggested DNA compression at the Y-junction rather DNA bending.

Another piece of evidence came from the release of the packaging-arrested Y- or X-branched DNA structures by the portal-bound T4 gp49 Holliday junction resolvase [138]. This resolvase function is probably essential in vivo because the packaging machine would often encounter such substrates in the newly synthesized concatemeric DNA that is also heavily branched, causing the stalling of the machine. Lindsay and colleagues simulated such a scenario in vitro by using the GFP-labeled portal protein and the Y-DNA substrates [125]. Additionally, they used dye-labeled gp17 in place of the label on the Y-DNA. In both cases, packaging was stalled at the Y-junction, and FRET measurements showed a reduction in the distance between the labels. This reduction and the stalled packaging were reversed by the addition of purified gp49 resolvase enzyme. These data also suggested DNA compression and further supported the domain movement model that is central to the electrostatic-force-driven DNA-packaging mechanism [31]. 

Lindsay suggested that terminase compression of DNA would transiently cause the local disruption of base-pair stacking and conversion to A-form DNA [137], which would then return to B-form DNA, resulting in a “spring-like” DNA movement. The release of the DNA-intercalated dyes such as ethidium bromide and YOYO-1 during translocation was considered as evidence for the disruption of base-pair stacking during translocation [139]. His subsequent studies showed that A-form DNA-RNA oligonucleotides are efficiently packaged, although, not surprisingly, the A-form double-stranded RNA oligonucleotides were not packaged [140]. In fact, this was the last report of his extensive investigations, in which he once again asserted the compression model and also argued against a “scrunch worm” model proposed by Steve Harvey, largely inspired by Lindsay’s A-form transition model. In the scrunch worm model [141], the terminase-DNA interaction would dehydrate the DNA in the motor channel, causing it to convert into an A-form with a ~23% length reduction. Rehydration back to B-form causes DNA elongation and ~2.5 bp translocation into the capsid for each ATP hydrolysis, which agreed with the experimentally determined ~2.5 bp motor step size of the phi29 packaging motor that is coupled to each ATP hydrolysis event [142]. 

The potential involvement of A-form DNA intermediate in translocation was evaluated by single-molecule optical tweezer studies [67]. A DNA construct was engineered by inserting a ~2 kb synthetic G-C rich A-philic sequence inserted into the middle of a ~9 kb “normal” B-form (non-A-philic) plasmid DNA sequence, and the packaging dynamics were measured using the T4 motor. Because the A-philic DNA is expected to transition more easily into A-form, the motor velocity is expected to increase when the motor encounters the A-philic sequence. However, no significant differences in the motor velocity were observed when compared to packaging the B-form DNA. This was also true even when a high force of 30 pN was applied where the differences in the ease of B to A transition are predicted to be the highest. Additionally, no significant alterations in motor dynamics, such as pausing, were observed between the two forms of the DNA or in general between different DNA sequences. The relatively high degree of variability in T4 motor velocities observed in previous studies are most likely of a stochastic nature but not due to differences in sequence. 

Recent cryo-EM structural studies of the phage phi29 packaging motor [143] and single-molecule tweezer and TIRF studies of the phage T4 motor [124] implicate DNA structural changes during translocation. However, it is not known whether these relate to force generation. In the cryo-EM structure of the phi29 packaging machine, the motor subunits and DNA form a complex in which the motor subunits appear to conform to the shape of DNA helix and track the 5′-3′ strand [136,143]. The DNA in the motor channel is stretched and partially unwound or compressed in some places, significantly deviating from the canonical B-form DNA. This is consistent with the tweezer studies on the T4 motor that showed tight gripping of DNA in the presence of ATP, likely involving all five subunits of the motor [117]. The gripping is so tight that little slipping occurs at 5 pN of applied force. Even at 30 pN of force, though the slipping rate increases, the friction force of the bound subunits still apparently matches the applied force. 

Given the spatial constraints in the binding of the five motor subunits to DNA at the same time, and combined with the expectation that the same chemical elements are probably recognized by the DNA-binding groove, the only way that all five motor subunits can simultaneously bind to DNA is to wrap around it in a helical configuration, one subunit per ~2 bp and five subunits per ~10 bp or one helical pitch. The motor subunits, which might otherwise form a ring structure in the absence of DNA, must be flexible enough to adjust to a DNA helix during active translocation. This would introduce a degree of tension and asymmetry in both the pentameric motor and the helical DNA. Consequently, in the motor–DNA complex, the subunits bind to DNA with different strengths. Additionally, asymmetric interactions in the symmetry-mismatched portal vertex [33] would also probably provide additional flexibility to attain this configuration. 

#### 4.4.3. Continuous Burst

**Figure 7:**
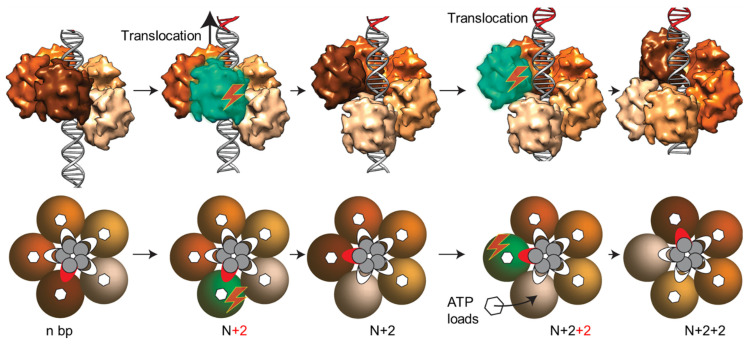
A model for the continuous burst packaging mechanism by the phage T4 DNA-packaging motor. The pentameric motor is shown to interact with DNA helix at different strengths, as depicted by increasing color intensity with increasing strength of the DNA grip. The top row shows surface views, and the bottom row shows spheres of the proposed DNA-binding domains of gp17. The firing subunit is shown in green, DNA helix in gray, ATP as hexagons, DNA grips as white patches, and the one with the strongest DNA grip as a red patch. “n” represents the motor prior to translocation, and “N+2” and “N+2+2” represent the motor when a subunit fires, causing 2 bp translocation. See the text for the details on the mechanism.

The above arrangement calls for a revision of our previous model, in which the motor subunits are thought to assemble as a ring structure (Figure 7). While this might be true in the absence of DNA, an actively translocating machine loaded with ATP would probably reorient the subunits in a helical shape in complex with the double helical DNA substrate. Consequently, one of the motor subunits (e.g., subunit 1) binds DNA with the highest affinity, and the rest would bind in a decreasing order because conforming to a DNA helix (which is also not perfectly symmetric) would induce a degree of strain in both the motor subunits and the DNA. Hence, subunit 5 would bind with the least affinity owing to the accumulated strain on the binding elements. This would also provide a mechanism as to which subunit is fired. We propose that subunit 1 with its strongest grip on DNA is triggered to fire ATP hydrolysis, resulting in the translocation of ~2 bp into the capsid (Figure 7). Subunit 2 then attains the strongest affinity, while subunit 1 in an ATP-free apo state with the least affinity to DNA reloads ATP. Subunit 2 then fires, causing another 2 bp of packaging, repeating the next cycle of the translocation, ATP-reloading, and DNA-gripping steps.

In the above model, ATP loading and ATP hydrolysis happen simultaneously in different subunits of the motor, and the hierarchical DNA interactions among the motor subunits are continuously remodeled as the motor tracks the DNA double helix. Consequently, because there would be no pause for ATP loading, the T4 motor translocates with a continuous burst and can attain high motor velocities. Its measured rate is up to >2000 bp/s, ~eight times faster than the phi29 motor [118]. The phi29 motor, on the other hand, takes a pause (dwell) after each burst cycle to reload the motor with ATP [73]. These periodic dwells that follow bursts in each cycle slow down the motor. 

This model is consistent with recent studies in which flexible coordination of the T4 motor was observed [124], as opposed to strict coordination of the phi29 motor. Mutant T4 motors were assembled consisting of a mixture of wild-type and Cy-3-labeled, ATPase-defective (dead) gp17 subunits. Single motors containing a defined number of dead subunits (0, 1, 2, etc) were selected by counting the number of Cy-3 labels. Engagement of individual motors with Cy-5 labeled DNA and their encapsidation behaviors were then examined in real time [124]. These measurements showed that the T4 motor can tolerate one, two, or even three dead subunits, although the defective motors spend less time in the packaging mode and are less efficient in encapsidating the oligonucleotide substrates. Whenever an inactive subunit encounters DNA, unable to hydrolyze ATP, the mutant subunit pauses and undergoes microslips such that the DNA grip is adjusted and realigned such that another wild-type subunit takes over and restarts ATPase firing and DNA translocation. These micropauses and microslips occur at fast timescales and could not be resolved by TIRF, though they are reflected in reduced packaging velocity and encapsidation efficiency. Furthermore, on some occasions, the pauses are long and lead to unpackaging, as has been observed when one or more motor subunits are in the apo state when ATP concentration is limiting [122]. Thus, the continuous burst model is overall consistent with a large number of structural, molecular, and single-molecule data from T4 and phi29 motors. 

The evolution of a flexible T4 motor with a continuous burst might allow phage T4 to package its 171 kb DNA in about the same amount of time as the phi29 motor takes to package its 19 kb DNA. In essence, it appears that there might be two classes of packaging motors: strictly coordinated, slow motors (phi-29 type), and flexible, fast motors (T4-type). However, the underlying basic translocation mechanism might be well conserved in phages and viruses. The former type is coordinated at the whole motor level whereas the latter type is controlled at the individual subunit level such that the basic tasks of translocation, namely ATP loading, DNA gripping, and ATP hydrolysis, occur without strict dependence on its neighbor. While this mechanism might lead to more-frequent pauses and slips owing to a lack of tight coordination, its ability to readjust the DNA grip or skip subunits when needed would allow T4 phage to package fast and to more easily overcome obstacles encountered when packaging a highly metabolically active, recombinogenic, and branched concatemeric genomic DNA.

## 5. Perspective

It has been 42 years since Lindsay and one of us (Rao) began exploring the T4 packaging machinery, inspired by his deep interest in this problem. Substantial progress has been made over these years. The structures of most of the components have been determined, and their biochemical functions and genetic phenotypes have been well characterized. Single-molecule assay systems have been developed that uncovered the dynamics of individual motors in real time. The mechanism is now narrowed to basically one model, and its details are emerging. Some of the basic knowledge is being translated to vaccines and gene therapies. Lindsay’s contributions span the entire spectrum of this fascinating phage biology, using the “beautiful” T4 as a model specimen.

While Lindsay’s contributions were numerous and broad, the one question that received his most attention was how DNA might be actively participating in the packaging mechanism, a question that hardly received anyone else’s attention. On the basis of his early genetic data, Lindsay proposed a DNA supercoiling model for packaging in 1978 [144] and continuously refined it over the years as his approaches became more and more precise and sophisticated. The field has learned a great deal from his creative and out-of-the box ideas, and he passed on his unique perspectives to numerous students, research fellows, collaborators, and colleagues all over the world.

Sharing a bench with Lindsay for 9 years was a major highlight of my career. Through natural osmosis, I received a bit of his passion, creativity, and dedication, which continue to serve as sources of inspiration for me. The particular delight that Lindsay took in working on the bench, virtually every single day like a graduate student, inspires me and many others who had the privilege and good fortune to be part of his research program. 

Perhaps the best way to honor Lindsay’s memory is to continue to strive to tease out the packaging mechanism, particularly the DNA structural transitions, at the highest resolution possible. While we have a good plausible model, it is still speculative. The emerging cryo-EM and single-molecule biophysics approaches, combined with the classic genetic and biochemical approaches, now provide us with powerful new opportunities to examine the packaging machine in action. We might be able to resolve the DNA structural and motor domain movements and transitions at near-atomic resolution. Equally fascinating is how the translocation mechanism is intimately connected to genome compaction inside the shell and how it seamlessly unravels and flows into a new host during infection, which is nothing short of a “miracle”.

## Data Availability

Not applicable.
